# The sero-prevalence and sero-incidence of African horse sickness and equine encephalosis in selected horse and donkey populations in Zimbabwe

**DOI:** 10.4102/ojvr.v84i1.1445

**Published:** 2017-05-10

**Authors:** Stuart J.G. Gordon, Charlotte Bolwell, Chris W. Rogers, Godfrey Musuka, Patrick Kelly, Alan Guthrie, Philip S. Mellor, Christopher Hamblin

**Affiliations:** 1 Institute of Veterinary, Animal and Biomedical Sciences, Massey University, New Zealand; 2 International Center for AIDS Care and Treatment Programs, Mailman School of Public Health, Columbia University, United States; 3 Ross University School of Veterinary Medicine, St. Kitts, West Indies; 4 Equine Research Centre, Faculty of Veterinary Science, University of Pretoria, South Africa; 5 International Research Centre, The Pirbright Institute, United Kingdom

## Abstract

Sentinel herds and samples submitted by private equine practitioners were used to determine the sero-prevalence and sero-incidence of African horse sickness virus (AHSV) and equine encephalosis virus (EEV) in horse and donkey populations in the Highveld region of Zimbabwe. The sero-prevalence and sero-incidence of antibodies against these viruses were determined using the competitive enzyme-linked immunosorbent assay (ELISA) for the detection of serum antibodies. In donkeys, the median sero-prevalence of AHSV antibodies, across the three rainy seasons under study, was 75% (inter quartile range [IQR] 67–83), with a seasonal median sero-incidence of 45% (IQR 40–63). In horses, the median sero-prevalence of EEV antibodies was 63% (IQR 21–73), with a median seasonal sero-incidence of 10.5% (IQR 10–14), while in donkeys the median sero-prevalence of EEV antibodies was 80% (IQR 67–90), with a median seasonal sero-incidence of 50% (IQR 40–60). This study highlighted the significant levels of exposure of donkeys to AHSV and horses and donkeys to EEV in Zimbabwe despite equine encephalosis remaining unreported by Zimbabwean veterinarians to date. Most seroconversions in sentinel herd animals to AHSV and EEV occurred towards the end of the rainy season in March, April and May corresponding to the time of the year when the *Culicoides* vectors are in high abundance. In order to determine the clinical significance of these infections, blood and spleen samples, submitted by private equine veterinary practitioners over a 5-year period, from horses showing characteristic clinical signs of African horse sickness were tested for the presence of viral antigen using the antigen capture ELISA. The median sero-prevalence of AHSV antigen in horses recorded from these samples was 38% (IQR 33–88). The predominant AHSV antigen from these samples was serotype 7 (33%) followed by serotype 2 (26%) and serotypes 4 and 8 (16% each). African horse sickness virus serotypes 3 and 9, identified in this study, had not been previously reported in Zimbabwe.

## Introduction

African horse sickness (AHS) and equine encephalosis (EE) are significant horse diseases in Zimbabwe caused by arthropod-borne viruses belonging to the Reoviridae family, genus *Orbivirus*. Mortality rates of up to 95% because of AHS have been recorded in unvaccinated horses in South Africa (Coetzer & Guthrie 2004). Namibia lost an estimated 10% of its horse population to AHS in 2011 (Liebenberg et al. 2016). The donkey is resistant to both AHS and EE but is considered to be an ideal sentinel to determine the prevalence and distribution of AHS virus (AHSV) and EE virus (EEV) through the detection of specific antibodies resulting from natural infection (Howell et al. 2002).

In contrast to AHS, EE is perceived as an emerging infectious disease and was first identified in 1967 by Erasmus et al. (1970) in horses in South Africa. EE has also been reported in Botswana, Kenya, Namibia, Zimbabwe (Coetzer & Guthrie 2004; Grewar et al. 2015; Paweska et al. 1999), Ethiopia, Ghana, The Gambia (Oura et al. 2012) and Israel (Wescott et al. 2013). Infections of horses with the EEV manifest typically as subclinical or mild disease (Crafford et al. 2011). Crafford (2002) described EE morbidity rates in horses in South Africa exceeding 74% with mortality rates up to 5%.

The distribution of AHS and EE in southern Africa seems to be governed by a number of factors including the prevalence and seasonal incidence of the major arthropod vector(s), which are certain species of *Culicoides* biting midges (Diptera: Ceratopogonidae) (Liebenberg et al. 2016; Mellor & Hamblin 2004). This suggests that the incidence of these diseases will have the same seasonality as the vector species. Gordon et al. (2015) found the greatest diversity and highest median counts of *Culicoides* to be in the central Highveld region of Zimbabwe where climatic and geophysical conditions favoured optimal breeding and larval development. Although the vector competence of most *Culicoides* species in Zimbabwe has not been studied in detail, Gordon et al. (2015) isolated Orbiviruses from *Culicoides imicola* which included EEV. In Zimbabwe, Blackburn and Swanepoel (1988) and Musuka and Kelly (2000) reported that AHS was diagnosed in late summer and autumn, which corresponds to high recorded numbers of the *Culicoides* vector (Gordon 2010).

The objectives of this study were to determine the sero-prevalence and sero-incidence of AHSV and EEV in horses and donkeys from sentinel herds established around Harare and Bulawayo in Zimbabwe. The serotypes of AHSV in animals from the sentinel herds and from the samples of suspected clinical cases in horses were also determined.

## Materials and methods

### Identification of sampling herds

Donkeys and horses on selected farms around the cities of Bulawayo and Harare, in Zimbabwe, were sampled to determine the sero-prevalence and sero-incidence of AHSV and EEV antibodies. Both locations lie in the central Highveld of Zimbabwe and experience climatic conditions that favour the abundance of the *Culicoides* vector(s). Farms were chosen based on a minimum number (> 6) of adult (> 18-month-old) donkeys or horses present and on-farm owner compliance. The farms included in the study were all of similar size and contained soil types, vegetation types and husbandry practices typical of farms in the Highveld of Zimbabwe.

### Determining the sero-prevalence of African horse sickness virus and equine encephalosis virus antibodies in sentinel herds

Venous blood samples (5 mL) were taken from all horses on participating farms using ethylene diamine tetra-acetic acid (EDTA) vacutainers and tested for EEV antibodies to determine the sero-prevalence of EEV in these herds. Blood samples were also taken from all donkeys on participating farms and tested for antibodies against AHSV and EEV to determine the sero-prevalence of AHSV and EEV in these donkey herds. Due to the widespread use of the live, attenuated, AHS vaccine (Onderstepoort Biological Products^®^) in horses in Zimbabwe, the sero-prevalence of AHSV in horses could not be determined as AHSV-specific antibodies may have been derived from vaccine viruses. The detection of antibodies against AHSV was determined using a competitive enzyme-linked immunosorbent assay (ELISA) following the methodology described by Hamblin et al. (1992). The detection of antibodies against EEV was determined using an ELISA following the methodology described by Crafford (2002) and subsequently published by Crafford et al. (2011).

### Determining the sero-incidence of African horse sickness virus and equine encephalosis virus antibodies in sentinel herds

Sentinel herds of donkeys and horses were established, at the same farms used to determine the sero-prevalence of antibodies to these two viruses during three rainy seasons (1999–2002). These sentinel herds were used to determine the sero-incidence of AHSV antibodies in donkeys and EEV antibodies in horses and donkeys by determining the rates at which sentinel animals seroconverted to AHSV or EEV. The sentinel herds were established using healthy adult horses and donkeys of both sexes that tested as seronegative against AHSV antibodies (donkeys) and EEV antibodies (horses and donkeys). A total of three horse sentinel herds were established around Harare and six around Bulawayo. Two donkey sentinel herds were established on farms around Harare and one around Bulawayo. Venous blood samples from the animals in the sentinel herds were taken using EDTA vacutainers and tested for antibodies against AHSV and EEV every 2 weeks, during each of the three rainy seasons (November–April), until seroconversion was detected.

### Private equine veterinary practitioners’ samples

Private equine veterinary practitioners, working within the Highveld region of Zimbabwe, were asked to monitor for suspected clinical cases and deaths from AHS in horses located within their veterinary practice catchment area over a 5-year period from 1998 to 2003. During this period, spleen samples, blood samples and combined spleen and blood samples were submitted by private equine veterinary practitioners from horses suspected to have died from or suffering from clinical AHS. No clinical cases of EE in horses or donkeys had been reported by practising veterinarians in Zimbabwe to that time although all submitted samples were tested for both AHSV and EEV. The blood samples submitted were collected in EDTA from live patients and the spleen tissue from dead patients following the recommendations outlined by Hamblin et al. (1992). Some practitioners submitted both ante-mortem blood samples and post-mortem spleen samples from fatal clinical cases.

All submitted blood and spleen samples were washed and lysed using techniques recommended by Hamblin et al. (1992) and Musuka and Kelly (2000). A serogroup specific, indirect, sandwich, antigen capture ELISA developed by Hamblin et al. (1991) and Crafford et al. (2003) was used to detect the presence of AHSV and EEV antigens respectively in these samples.

Virus isolations were made by intracerebral inoculation of 2-day-old suckling mice (CD-1, UK) and intravenous inoculation of 11-day-old embryonated hens’ eggs (IAH, UK). In each case, 100 μL of a 1 in 10 dilution of lysed blood or homogenated spleen was inoculated. Brains from dead mice and hearts from dead embryos were homogenised as a 1 in 10 suspension and serially blind passaged up to three times in baby hamster kidney (BHK) tissue culture cells to amplify any virus present and to adapt them to growth in cell culture.

### Viral serotyping

The AHS viruses were typed by virus neutralisation, using microtitre plates and BHK cells, following methods similar to those described by Howell (1962). Virus neutralisation tests were performed on viruses isolated from EDTA blood and spleen samples submitted by private practitioners as well as those isolated from EDTA blood samples from sentinel herd animals 2 weeks before they seroconverted. Assaying the blood samples collected before animals seroconverted maximised the chance of isolating free virus. AHSV positive sera, from sentinel animals that had seroconverted but were negative for virus isolation, were serotyped in neutralisation tests using defined serotype-specific AHS viruses, using microtiter plates and BHK cells, following methods modified from Blackburn and Swanepoel (1988).

### Statistical analysis

Data were examined using simple descriptive statistics. Data were tested for normality using the Shapiro–Wilk test. Non-parametric data are presented as median and inter quartile range. The effect of season on prevalence was tested using a Kruskal–Wallis test. All analyses were performed using Stata IC v12 (College Station, TX, USA). The level of significance was set at *p* < 0.05.

## Results

### Sero-prevalence and sero-incidence of African horse sickness virus and equine encephalosis virus antibodies

Serum decanted from blood samples collected from 279 horses was tested for EEV antibodies over the three rainy seasons to determine the sero-prevalence of EEV in these herds. The small per farm sample size (22; inter quartile range [IQR] 14–28) provided a large between-farm variation in the sero-prevalence of EEV (Table 1). The highest prevalence was recorded during the second rainy season (2000/2001) although no significant difference between years in sero-prevalence was found (*p* = 0.059). The median sero-prevalence of EEV across the three rainy seasons under study in the horses sampled was 63% (IQR 21–73).

**TABLE 1:** The sero-prevalence of antibodies against equine encephalosis virus in horses in Zimbabwe, during three rainy seasons for the period 1999–2002.

Season	Number of farms	Number of horses bled	Number seropositive for antibodies against EEV	Sero-prevalence of EEV (%)	
				Median	IQR
1999/2000	6	150	39	28	11–62
2000/2001	3	74	55	76	70–70
2001/2002	3	55	36	64	29–100
All years	-	279	130	63*	21–73

EEV, equine encephalosis virus; IQR, inter quartile range.

* Differences between seasons (*p* = 0.059).

Serum decanted from blood samples collected from 155 donkeys was tested for antibodies against AHSV and EEV to determine the sero-prevalence of AHSV and EEV (Table 2). The median sero-prevalence of AHSV and EEV across the three rainy seasons under study was 75% (IQR 67–83) and 80% (IQR 67–90), respectively (Table 2).

**TABLE 2:** The sero-prevalence of antibodies against African horse sickness virus and equine encephalosis virus in donkeys in Zimbabwe during three rainy seasons for the period 1999–2002.

Season	Number of farms	Number of donkeys bled	Number seropositive for antibodies against AHSV	Sero-prevalence of AHSV(%)		Number seropositive for antibodies against EEV	Sero-prevalence of EEV (%)	
				Median	IQR		Median	IQR
1999/2000	2	69	42	64	60–67	30	53	39–67
2000/2001	1	35	30	86	-	30	86	-
2001/2002	3	51	40	80	69–83	45	90	78–100
All years	-	155	112	75	67–83	105	80	67–90

AHSV, African horse sickness virus; EEV, equine encephalosis virus; IQR, inter quartile range.

A total of six horse sentinel herds were established over three rainy seasons to determine the sero-incidence of EEV antibodies in horses. The sentinel herds consisted of a total of 76 horses and a median sero-incidence of EEV of 10.5% (IQR 10–14) was recorded.

One to two donkey sentinel herds were established during each rainy season under study to determine the sero-incidence of AHSV and EEV antibodies in donkeys. The sentinel herds consisted of a total of 18 donkeys, with each sentinel herd in each rainy season comprising 4–5 donkeys. A median sero-incidence of 45% (IQR 40–63) and 50% (IQR 40–60) was recorded for AHSV and EEV, respectively.

### Private equine veterinary practitioners’ samples

During a 5-year period from 1998 to 2003, 22 spleen samples, 92 EDTA blood samples and 11 combined spleen and EDTA blood samples were submitted by private equine veterinary practitioners from 125 horses suspected to have died from or suffering from clinical AHS (Table 3). Based on antigen detection from samples submitted by private practitioners, there was an overall median sero-prevalence of AHSV antigens in horses of 38% (IQR 33–88) (Table 3). Three of the 125 samples tested positive for EEV antigens.

**TABLE 3:** Horse samples submitted by private equine veterinary practitioners for the detection of African horse sickness virus antigens.

Season	Number of samples submitted				Number positive for AHSV	Prevalence of AHSV (%)
	Blood	Spleen	Blood and spleen	Total		
1998/1999	38	16	9	63	52	83
1999/2000	30	1	1	32	12	38
2000/2001	7	2	1	10	10	100
2001/2002	11	1	0	12	4	33
2002/2003	6	2	0	8	2	25
**Totals**	**92**	**22**	**11**	**125**	**80**	**-**

Median(IQR): 38(33–83).

AHSV, African horse sickness virus; IQR, inter quartile range.

### Viral serotyping

The predominant AHSV serotype identified from private equine veterinary practitioners’ horse spleen and blood samples and blood samples from donkey sentinel herd animals showing seroconversion was serotype 7 (19/57, 33%) followed by serotype 2 (15/57, 26%), serotypes 4 and 8 (9/57, 16% each) and serotype 6 (3/57, 5%). Serotypes 3 and 9 (1/57, 2%) were identified for the first time in Zimbabwe. Attempts to serotype EEV were unsuccessful.

## Discussion

This study recorded a high median sero-prevalence of antibodies against AHSV in selected donkey herds and a high median sero-prevalence of antibodies against EEV in selected horse and donkey herds in the Highveld region of Zimbabwe. A sero-prevalence for antibodies against EEV of 56% – 87% in horses and donkeys and a sero-prevalence of AHSV of 44% – 64% in horses were previously reported in Zimbabwe (Blackburn 1982; Blackburn, Searle & Phelps 1985; Blackburn & Swanepoel 1988; Musuka 1999; Musuka & Kelly 2000; Paweska et al. 1999). Howell et al. (2008) found the seasonal sero-prevalence of antibodies against EEV in South Africa to vary between 3.6% and 34.7%.

Because of the annual polyvalent prophylactic immunisation against AHSV practised in horses in Zimbabwe at the time of this study, serotype-specific antibody derived from immunisation, natural challenge or the transfer of colostrum could not have been distinguished by conventional serum viral neutralisation assays (Howell et al. 2008). Since sero-epidemiological surveys would, therefore, have been inconclusive in establishing seroconversion to natural AHSV challenge, horse sera were not tested for antibodies against AHSV in this study. No donkeys in this study had received prophylactic immunisation against AHSV meaning that antibodies against AHSV were most likely derived from natural challenge.

The median seasonal sero-incidence of EEV antibodies was found to be higher in donkey sentinel herds than in horse sentinel herds. This could be because of the AHS vaccine used in horses conferring some form of protection against EEV in this species but may also reflect the fact that donkeys are rarely stabled at night in Zimbabwe and are thus more at risk of exposure to the *Culicoides* vector(s) than are most horses. Furthermore, the high incidence of AHSV and EEV antibody detection in donkeys should be interpreted with caution as the sentinel herd sample size of donkeys in this study was small.

The prevalence and incidence rates, however, show that horses and donkeys face a significant rate of exposure to both AHSV and EEV in Zimbabwe. Previously, EE in southern Africa was thought to be mainly distributed in South Africa and Botswana (Paweska et al. 1999), and so these results have emphasised the importance of this disease in horses and donkeys in Zimbabwe.

The sentinel herds were situated in the Highveld of Zimbabwe, which is characterised by high rainfall and temperature that favour the abundance of the *Culicoides* vector(s). The sero-incidence of AHSV and EEV antibodies in sentinel herds at some of the farms closely matched the number of *Culicoides* vectors trapped per night at these farms, as reported in previous studies by Gordon (2010) and Gordon et al. (2015).

Most sero-conversions to AHSV and EEV in this study occurred from January to April, 3–4 months after the beginning of the annual rainy season. This delay reflects the time taken for vector numbers to increase after the beginning of the annual rains and the time for a viraemia to develop in animals, which then act as sources of infection for the vectors (Mellor 1990). The clinical cases and samples submitted by practitioners also displayed this apparent seasonal latency. This temporal pattern of a delay in sero-conversions has been previously reported by Blackburn and Swanepoel (1988) and Musuka and Kelly (2000) where most cases of AHS were diagnosed in late summer and autumn coinciding with the end of the Zimbabwean rainy season in March to May. However, further studies utilising rainfall data would be required in order to demonstrate an association between rainfall and the sero-incidence of AHSV and EEV.

The predominant AHSV serotype recorded in this study was serotype 7, which was identical to the findings reported in the 1980 outbreak of AHS in the north west of Harare (Blackburn & Swanepoel 1988). In the current study, AHSV serotypes 3 and 9 were identified for the first time in Zimbabwe. Despite the detection of EEV antigen in three of these samples submitted by private practitioners, no serotypes for EEV could be identified. In a study on EE in thoroughbred yearlings in South Africa, serotypes 1 and 6 were most frequently identified, while the remaining serotypes 2, 3, 4, 5 and 7 were only identified sporadically in localised infections of EE affecting individual horses (Howell et al. 2008).

## Conclusion

This study highlighted the significant levels of exposure of horses and donkeys to AHSV and EEV in Zimbabwe. The sero-incidence of AHSV and EEV infection and the clinical cases of AHS were highest at the end of the rainy season in March and April coinciding with peak abundance of the *Culicoides* vector(s). The main AHSV serotype responsible for causing clinical AHS in the blood and spleen samples submitted by private equine veterinary practitioners and blood samples from sentinel herd animals showing seroconversion was serotype 7, corroborating previous AHSV identification research findings in Zimbabwe. This study identified the presence of AHSV serotypes 3 and 9 in Zimbabwe for the first time.

African horse sickness continues to be a serious problem in Zimbabwe despite the extensive vaccination programme conducted in horses across the country at the time of this study. As EE is largely undiagnosed by equine veterinary practitioners in Zimbabwe, the high reported sero-prevalence and sero-incidence of this disease will hopefully alert Zimbabwean veterinarians to the possibility of its occurrence in both horses and donkeys.
